# To What Extent Can We Extrapolate Proteochemometric Models: A Case Study for the SLC6 Transporter Family

**DOI:** 10.1002/minf.70043

**Published:** 2026-07-28

**Authors:** Uday Abu‐Shehab, Gerhard Ecker

**Affiliations:** ^1^ Department of Pharmaceutical Sciences University of Vienna Vienna Austria

**Keywords:** proteochemometrics, SLC6 family

## Abstract

Proteochemometrics (PCM) modeling combines protein and ligand information to create predictive models for biological activity. It aims to extrapolate information across targets, enabling its application in screening drug candidates across a whole family of proteins. In this study, we investigate the ability of PCM models to extrapolate information from data‐rich proteins to data‐poor ones and present a series of PCM models for the SLC6 transporter family showing reasonable performance (Q2 values up to 0.79). Moreover, feature importance analysis pointed towards residue position A173 in hSERT, corresponding to G149 and G153 in hNET and hDAT, respectively, to be relevant for subtype selectivity. However, examining the impact of different data splits on model validation metrics highlights potential over‐optimism when only considering target stratification splits. Target stratification split only maintains the train/test ratio across all targets, preserving per target balance without considering chemical similarity. However, when performing leave‐one‐transporter‐out studies, a considerable drop in performance was observed. This points towards the need for more complex technologies to exploit the potential of PCM and identify new drug candidates.

## Graphical abstract

Proteochemometrics models for the SLC6 transporter family achieve reasonable predictive performance and highlight a selectivity‐relevant residue (SERT A173/NET G149/DAT G153). However, performance drops in leave‐one‐transporter‐out validation, indicating that true extrapolation to unseen transporters remains limited and current evaluation can be over‐optimistic.

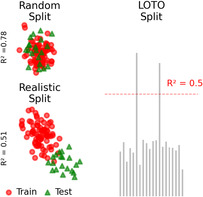

## 1 Introduction

The Solute Carrier Transporter Family 6 (SLC6) represents an important group of transporters within the large family of Solute Carriers (SLCs), playing an essential role in transporting neurotransmitters, amino acids, and osmolytes across cell membranes [1]. Comprising several subfamilies, SLC6 transporters are fundamental to an array of physiological processes and neural signaling pathways [2]. A prominent example is the monoamine transporter subfamily (MAT) comprising the serotonin‐, norepinephrine‐, and dopamine transporters, which have been a major area of interest for their therapeutic effects in treating neuropsychiatric disorders [1, 2]. Additionally, the SLC6 family includes other subfamilies, such as the GABA, amino acids, and nutrient transporter subfamilies [2]. However, these subfamilies are considerably less explored than the MAT subfamily [1, 3].

Proteochemometrics (PCM) modeling is a technique that builds on quantitative structure–activity relationship (QSAR) models by incorporating both protein and molecular information [4]. Previous work demonstrated several advantages of PCM modeling compared to traditional QSAR methods, such as providing more reliable predictions [5–7]. More importantly, PCM modeling has the potential to extrapolate chemical information across proteins, especially when transferring data from data‐rich proteins to data‐poor ones. This is particularly useful to explore understudied protein members or even screen drugs against orphan targets [4].

PCM models are typically validated using both internal and external validation methods [4]. However, several factors influence the reliability of these validations, including how the dataset is split into training and testing sets and any imbalances in the dataset [6]. For instance, a random split often leads to over‐optimistic results, particularly when highly similar compounds are in both the training and test sets. Additionally, evaluating the entire test set with a single performance metric (like R2 or MCC) can be skewed by imbalances in the data available for different targets. This can be highly misleading as it misrepresents the model's ability to extrapolate to new target spaces. These challenges highlight the importance of a careful and thoughtful evaluation strategy for PCM models.

Bongers et al. [8] recently demonstrated the potential of PCM models, specifically for the SLC6 family, by identifying five novel inhibitors for the norepinephrine transporter (SLC6A2). The five identified inhibitors had, on average, a Tanimoto similarity coefficient of 0.38 (the lowest is 0.25), indicating novelty in the identified inhibitors. The authors also demonstrated that PCM modeling outperforms traditional QSAR methods, where the model was trained on the norepinephrine transporter data only (>0.1 *R* ^2^ improvement on the external test set) [8]. However, given that the norepinephrine transporter is among the most studied SLC6 members for its clinical significance, our focus here will be on the understudied SLC6 members that lack sufficient data.

In this paper, we trained PCM models on bioactivity data for 13 SLC6 members across various organisms. Our results demonstrated that a target‐stratified data split led to over‐optimistic models, whereas the realistic split recommended by Martin et al. [9] resulted in more conservative and robust models, reducing the risk of overfitting. Target‐stratified split aims to maintain the train/test ratio across all proteins without considering the similarity between molecules. The realistic split, on the other hand, assigns the most dissimilar molecules of the dataset to the test set, mimicking a realistic screening scenario where the screened compounds may look very different compared to the training data. Furthermore, Leave One Target Out (LOTO) validation was investigated for the potential of PCM in identifying novel drug candidates for unexplored orphan targets, demonstrating that extrapolation to novel orphan targets remains a major challenge for PCM modeling.

## 2 Methods

### 2.1 Defining the Binding Pocket

**FIGURE 1:**
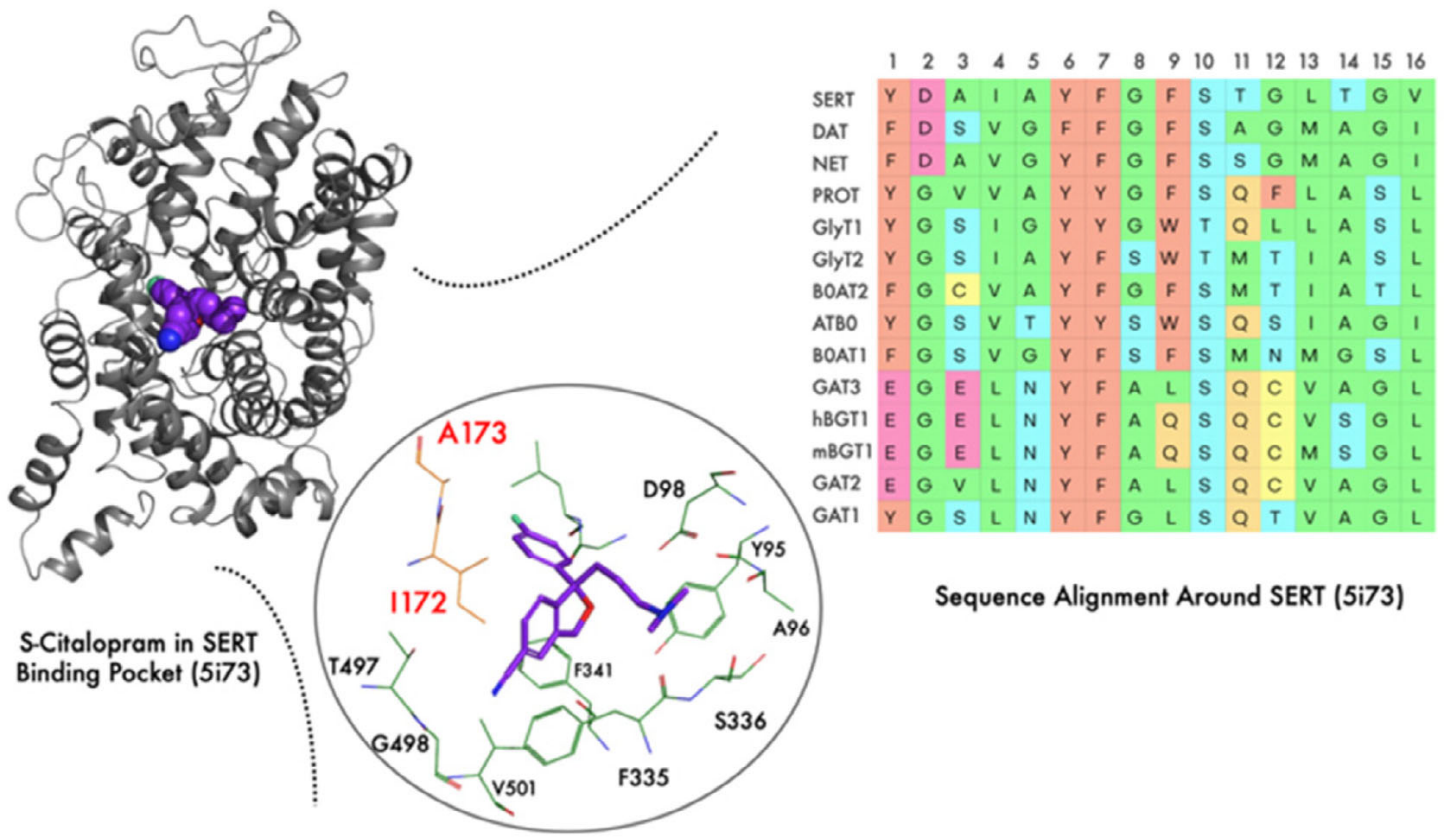
The binding pocket of the SERT cocrystallized with s‐citalopram and the sequence alignment of the binding pocket across the SLC6 family members.

We retrieved FASTA sequences of those SLC6 targets with IC50 data points in ChEMBL33 [10] and imported them into Molecular Operation Environment (MOE) software (version 2020.09) [11]. Consequently, the cocrystallized structure of the human serotonin transporter with S‐citalopram (PDB ID: 5i73) [12] was imported, and all sequences were aligned in the sequence editor. Afterwards, residues within a 5 Å radius around S‐citalopram were chosen, amounting to 20 residues. These 20 residues in hSERT and their corresponding residues in all SLC6 members were considered to represent the binding pockets, and all remaining residues were deleted. The residue position corresponding to Y176 in hSERT was identified to be conserved in all SLC6 members and was removed from the binding pocket since it remains constant across all SLC6 members. In addition, the residue positions corresponding to A96, S336, and L337 in hSERT were identified to be involved in backbone interactions with inhibitors of SLC6 members and subsequently eliminated from the binding pocket since backbone interactions are independent of the physiochemical properties of their corresponding side chains for which descriptors are calculated [13, 14]. This resulted in a binding pocket of 16 residues (Figure 1).

Further analysis showed that the binding pockets of a given SLC6 member across different organisms were identical. This was the case for all SLC6 members except for the betaine transporter (BGT1), having Val400 in the human isoform, hBGT1, and a corresponding Met400 in the mouse isoform, mBGT1. To avoid duplicating sequences, we deleted duplicates and labeled the remaining targets in an organism‐agnostic fashion. For instance, we used SERT as the label for the serotonin transporter across organisms instead of hSERT, mSERT, and rSERT for Homo sapiens, Rattus norvegicus, and Mus musculus, respectively. This required further curation steps of the dataset to prevent having identical data points with different endpoint labels, which is explained in detail in the dataset curation section.

### 2.2 Dataset Curation and Standardization

To create our dataset, we used the KNIME Analytics Platform 4.6.5 [15] to gather biological activity data from ChEMBL33 [10]. We obtained data for all available SLC6 members from various organisms, which initially amounted to 49 867 data points. We then curated the data and standardized the molecules. In addition, we added 28 ligands to our dataset that were previously tested on the SLC6A19 transporter and were not included in the ChEMBL database [16, 17, 18–19].

The dataset curation steps were designed to produce a high‐quality dataset suitable for regression modeling. Therefore, only data points with a standard_relation value of “=,” standard_units of “nM,” relationship_type of “D,” and standard_type of “IC50” were considered. Relationship_type is a target curation flag where the value “D” means that the bioactivity data is assigned directly to the target of interest (Other options could be “H” for homologous protein assignment). Only H. sapiens, M. musculus, and R. norvegicus were considered due to the abundance of available data on these organisms. Molecules were standardized using MolVS (version 0.1.1) [20] and RDKit (version 2022.09.1) [21] by sanitizing molecules, removing hydrogens, disconnecting metals, removing fragments, neutralizing charges, and removing stereochemistry. To maintain a homogeneous dataset, some molecules were removed for containing radioactive isotopes or silicon atoms. Additionally, data points were considered duplicates if they show identical standardized InChI codes, targets, and pChEMBL values and were thus eliminated.

In the dataset, all SLC6 members except BGT1 shared identical binding pockets across different organisms. We excluded duplicate entries to avoid conflicting data points, such as identical descriptors of the same molecule tested on H. sapiens and R. norvegicus serotonin transporters. The cleanup protocol included: (i) considering data from individual targets with identical binding sites across organisms, (ii) retaining data points tested against a single organism, (iii) retaining only H. sapiens data when a molecule was tested against it and another organism, and (iv) opting for R. norvegicus data over M. musculus data due to its larger dataset size when a molecule was tested on both. Finally, in the case of multiple pChEMBL values for the same InChI code/target pair, the data points were merged into one, the mean and standard error of the mean (SEM) of the pChEMBL values were calculated, and if SEM > 0.2, the data point was removed.

**FIGURE 2:**
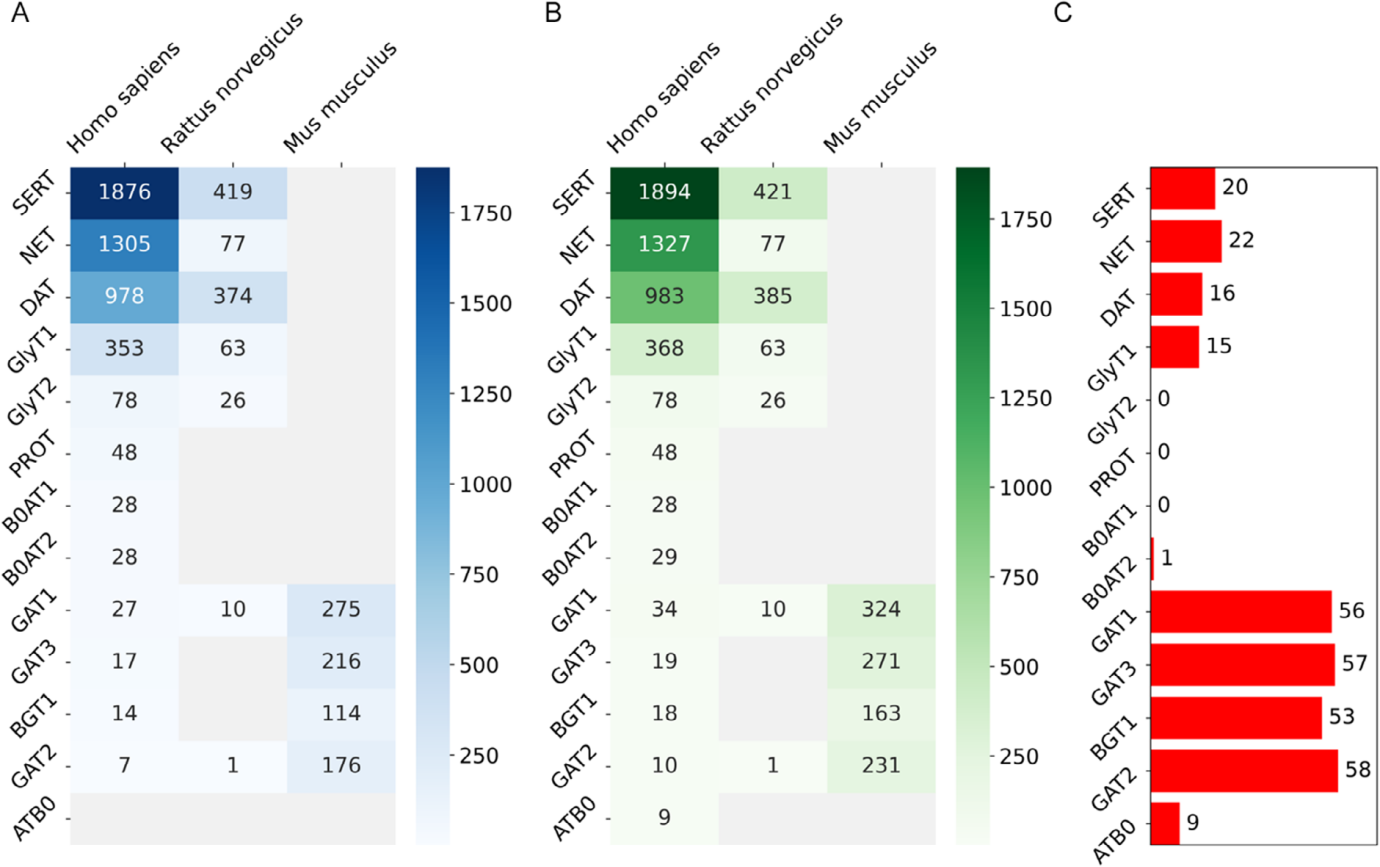
(A) Heatmap of the curated dataset showing the organisms versus targets. (B) Heatmap of the curated dataset showing organisms versus targets after adding the data points labeled “Outside typical range.” (C) Bar plot of the additional data points.

Further analysis of the data obtained from ChEMBL33 [10] revealed that numerous data points had a standard_value but no pChEMBL value. Although ChEMBL flagged these data points as “Outside typical range,” we manually calculated the pChEMBL values aiming at investigating the model's performance both with and without them. After curation, they amounted to 307 additional data points labeled as “Outside typical range,” 224 of which belonged to GABA transporters (Figure 2). The curation process resulted in 6510 data points for the first dataset with the automatically calculated pChEMBL values “auto” and 6817 data points for the second dataset with the manually calculated pChEMBL values “manual.”

To investigate assay coverage across the SLC6 family members, we assembled a ligand x target matrix of existing measurements. As illustrated in Figure S1, the overlap within the subfamilies of transporters is apparent. However, the overlap between the subfamilies is surprisingly negligible.

### 2.3 Molecular 2D Descriptors

The dataset was described using a set of physiochemical descriptors calculated by RDKit (version 2022.09.01) [21]. Any descriptor that showed a correlation greater than 95% with another descriptor was eliminated to avoid redundancy in the dataset. This resulted in 179 descriptors for the “auto” dataset and 180 descriptors for the larger “manual” dataset. The additional descriptor is “fr_C_S,” which represents the number of thiocarbonyl groups. This descriptor was not eliminated from the larger dataset because it no longer showed a significant correlation with other descriptors. For a full list of the used descriptors, please see Supporting Information.

### 2.4 Molecular 3D Descriptors

For the 3D representation of the molecules, we used 76 i3D VolSurf [22] descriptors calculated by the MOE software (version 2020.09) [11]. These descriptors transform the information present in the 3D molecular field maps into simpler and more interpretable descriptors such as molecular size, shape, polarity, and hydrophobic/hydrophilic regions. The 3D conformations of molecules were generated before the descriptor calculations using the force field MMFF94x in MOE.

### 2.5 Target Z‐scales Descriptors

Several studies have indicated superior performance when focusing on the specific amino acids that make up the binding site, instead of considering entire protein sequences. Therefore, we used Z‐scale descriptors [23] to describe the amino acids within the binding pocket individually. These Z‐scales account for 26 physicochemical properties of each amino acid,which are then reduced to five principal components using principal component analysis [23]. The first three components correspond to lipophilicity, volume/polarizability, and polarity [23].

### 2.6 Random Stratified Split

The dataset was split into 80/20 training/test sets and was stratified to account for the distribution of the different SLC6 members, i.e., each protein member contributed 80% of its data to the training set and the remaining 20% to the test set. The same random splitting principle was applied to construct the folds for cross‐validation. The splitting was performed without considering other factors such as pIC50 or chemical space distribution, essentially randomly assigning data to training and test sets within each protein member.

### 2.7 Realistic Split

To evaluate the accuracy of this model in actual virtual screening scenarios, we partitioned the data using the realistic split proposed by Martin et al. [9]. This method applies a hierarchical clustering procedure to the dataset based on Morgan fingerprint (bits = 1024, radius = 2). We used the reference implementation published by Martin et al. [9] without modifications. For datasets with fewer than 10,000 compounds, the implementation performs hierarchical clustering and cuts the dendrogram to produce an average cluster size of eight, which implicitly sets the number of clusters (∼N/8). Martin et al. reported that model performance is insensitive to changing cluster size.

The dataset is then sorted in descending order based on cluster size. The first 80% of the ordered dataset, representing the largest clusters of compounds with similar characteristics, is selected to form the training set, leaving small clusters or standing‐alone compounds to form the test set. Therefore, this approach emphasizes the novelty of the test set compared to the training set by reducing their similarity [9, 24]. This method splits the data solely based on molecular structures without considering target stratification. As a result, the test set was missing one target, namely SLC6A14, the Amino Acid Transporter B0,+ (ATB0,+). With its nine data points, this target was fully included in the training set. The split resulted in a training set with 208 clusters (5455 compounds) and a test set with 645 clusters (1362 compounds). Moreover, the same cluster‐based splitting principle was applied to construct the folds for cross‐validation.

### 2.8 LOTO

To evaluate the model's potential to predict responses for completely unseen target spaces, like orphan targets, we performed LOTO data splits. In this method, each target is sequentially considered a test set while the model is trained on the rest of the dataset, i.e., all other targets.

### 2.9 PCM Modeling

The random forest (RF) [25] and eXtreme Gradient Boosting (XGBoost) [26] algorithms were selected to train the PCM models. After feature selection, as described in the molecular descriptors section, models were trained and tuned using a grid search of hyperparameters to optimize performance during cross‐validation (Table S1).

### 2.10 Model Evaluation

After model training, predictions were evaluated using the Q2 [27] metric for the cross‐validation and R2 for the external test set. Additionally, the following performance metrics were calculated and reported for both the cross‐validation and test sets: mean absolute error (MAE), mean squared error (MSE), and root mean squared error (RMSE).

## 3 Results and Discussion

Upon evaluation of the PCM models on the two datasets, they exhibited varying performance (Table 1). The first dataset, “Auto,” contained only data points with pChEMBL values automatically calculated by ChEMBL (6510 data points). The second dataset, “Manual,” included additional data points with standard values but missing pChEMBL values as they were labeled “Outside Typical Range” by ChEMBL. Adding these manually calculated pChEMBL values increased the dataset by 307 data points and introduced a new protein target, ATB0, thereby expanding the biological space covered by the models. The addition of the curated data points statistically significantly improved model performance on both the validation (Q2 Paired‐t(7) = 6.01; *p*‐value < 0.001) and external test set (R2 Paired‐t(7) = 8.78; *p*‐value < 0.001). On the external test set, neither MSE nor MSE differed significantly between models trained on the two datasets (Auto vs. Manual; Paired‐t(7) = 1.25; *p*‐value = 0.25 for MSE and Paired‐t(7) = 1.26; *p*‐value = 0.25 for RMSE). While validation MSE and RMSE showed a small but statistically significant increase (Auto vs. Manual; Paired‐t(7) = 2.95; *p*‐value = 0.02 for MSE and Paired‐t(7) = 3.05; *p*‐value = 0.02 for RMSE), the absolute differences were negligible (ΔMSE = 0.03, ΔRMSE = 0.02 pChEMBL units).

**TABLE 1:** Performance of PCM models across datasets, descriptors, splits, and used algorithms.

Dataset^b^	Descriptors	AA desc.^a^	Split	Algorithm	Cross‐validation (10‐fold)				External validation set			
					Q2	MAE	MSE	RMSE	R2	MAE	MSE	RMSE
Manual	PC	z‐scales	Random	RF	0.77	0.51	0.44	0.66	0.78	0.51	0.44	0.67
Manual	PC	z‐scales	Random	XGB	0.79	0.48	0.40	0.64	0.79	0.48	0.42	0.65
Manual	PC	z‐scales	Realistic	RF	0.53	0.73	0.84	0.92	0.51	0.80	1.01	1.01
Manual	PC	z‐scales	Realistic	XGB	0.54	0.71	0.83	0.91	0.54	0.77	0.94	0.97
Manual	VSURF	z‐scales	Random	RF	0.66	0.62	0.64	0.80	0.69	0.62	0.62	0.79
Manual	VSURF	z‐scales	Random	XGB	0.66	0.62	0.64	0.80	0.65	0.63	0.67	0.82
Manual	VSURF	z‐scales	Realistic	RF	0.64	0.63	0.64	0.80	0.43	0.86	1.18	1.09
Manual	VSURF	z‐scales	Realistic	XGB	0.65	0.61	0.63	0.79	0.43	0.84	1.18	1.08
Auto	PC	z‐scales	Random	RF	0.72	0.51	0.44	0.66	0.72	0.49	0.42	0.65
Auto	PC	z‐scales	Random	XGB	0.74	0.47	0.40	0.64	0.74	0.47	0.39	0.63
Auto	PC	z‐scales	Realistic	RF	0.44	0.70	0.77	0.87	0.42	0.76	0.92	0.96
Auto	PC	z‐scales	Realistic	XGB	0.42	0.70	0.79	0.88	0.42	0.76	0.94	0.97
Auto	VSURF	z‐scales	Random	RF	0.63	0.59	0.59	0.76	0.57	0.62	0.65	0.80
Auto	VSURF	z‐scales	Random	XGB	0.61	0.60	0.62	0.78	0.51	0.66	0.74	0.86
Auto	VSURF	z‐scales	Realistic	RF	0.56	0.59	0.58	0.75	0.34	1.03	0.81	1.06
Auto	VSURF	z‐scales	Realistic	XGB	0.53	0.61	0.63	0.79	0.31	0.82	1.11	1.05

a AA desc.: Amino acids descriptors.

b Dataset: (i) Auto: automatic pChEMBL calculation by ChEMBL; (ii) Manual: manually calculated pChEMBL values, including those labeled as “Outside Typical Range.”

**FIGURE 3:**
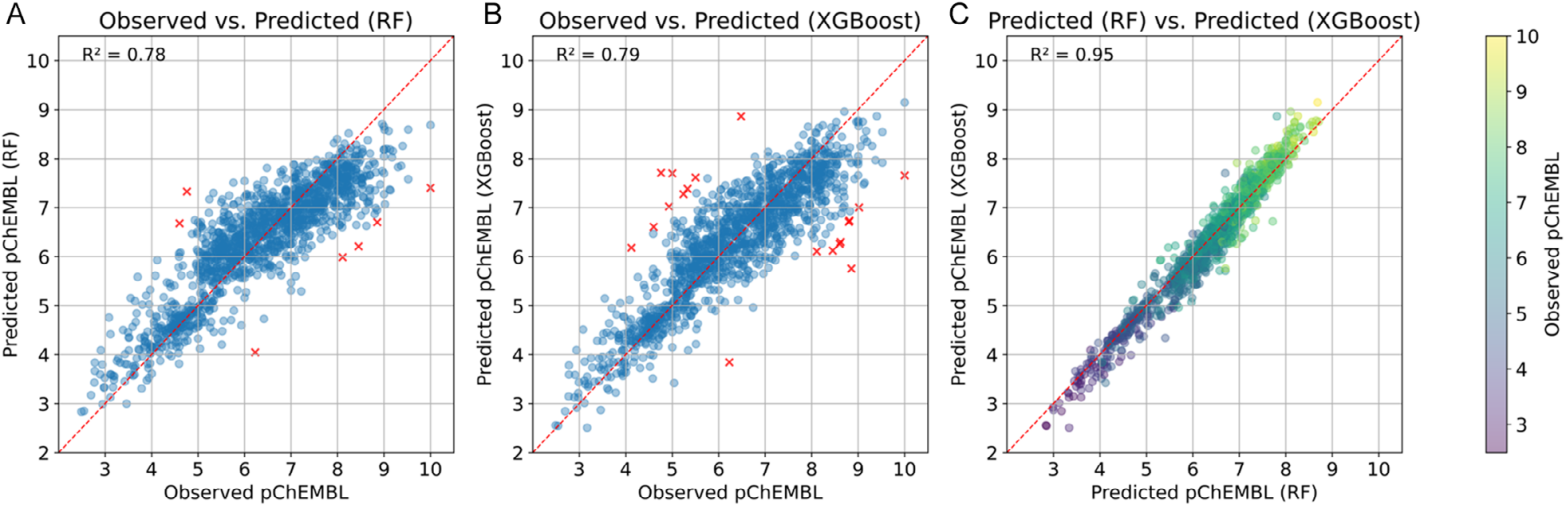
Scatter plot of observed versus predicted pChEMBL value for both RF (A) and XGBoost (B) models for the manual test set (Random split, PC, and z‐scales descriptors). Outliers are illustrated as red X marks. Subplot (C) plots predicted (RF) versus predicted (XGBoost) and illustrates the high degree of agreement between the predictions of the two algorithms, with no observable outliers. The color scale is the experimental pChEMBL value.

Predictions with an error greater than two log units were considered outliers. In our evaluation, the RF model showed seven outliers, while the XGBoost model showed 19 outliers (Figure 3). Notably, all seven outliers from the RF model were also present in the XGBoost model. Nine outliers in the test set were molecules already present in the training set but had activities on different targets, with a significant difference in activity between those targets. As an example, “CHEMBL571536” (Figure S2) is present in the test set with a pIC50 value of 4.76 against SLC6A2 (NET) and a predicted pIC50 value of 7.62. This error of 2.86 log units might be due to the presence of the same compound in the training set with a higher pIC50 value of 8.0 against SLC6A4. Nine out of the total 19 outliers follow this pattern. In general, a pattern of activity cliffs exists in the random split more often than in the realistic split. We define an activity cliff here as a pair of molecules (Tanimoto similarity coefficient > 0.8) with more than 30‐fold activity difference. For instance, in the manual dataset, the random split produces 337 activity cliffs in the train/test splits, compared to only nine in the realistic split.

**FIGURE 4:**
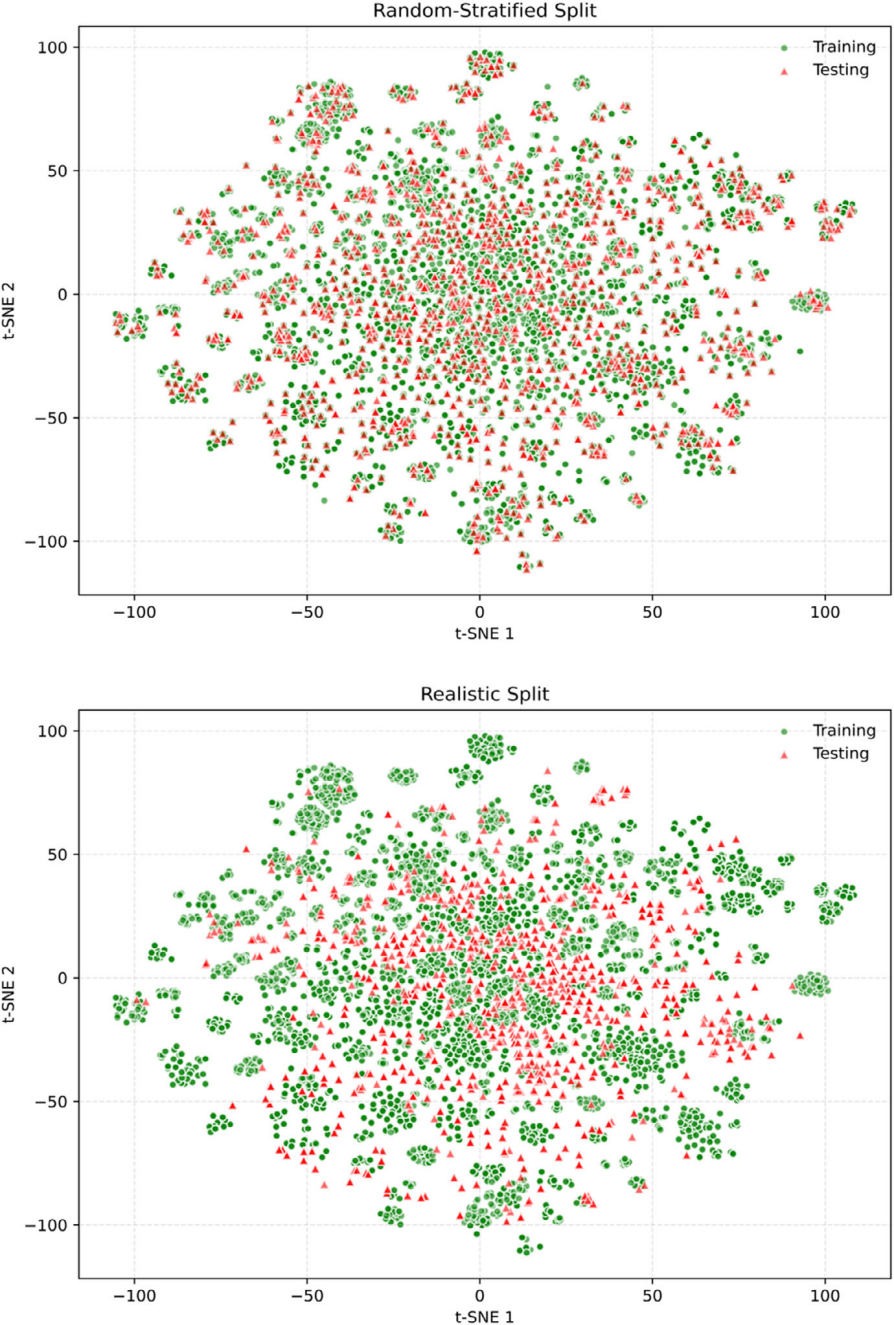
t‐SNE molecular visualization of the dataset according to the random‐stratified split (top), and the realistic split (down).

To determine if the PCM model is effective in virtual screening situations, we utilized the realistic split method recommended by Martin et al. [9]. This approach involves assigning the most dissimilar group of compounds to the test set to mimic screening scenarios where the screened chemical space is typically different from the one used to train the model. The difference between the test set in the realistic split and the random‐stratified split can be evident when using t‐SNE for chemical space visualization, as shown in Figure 4. In Figure 4A, one can observe the random nature of the test set as it covers the chemical space of the entire dataset, as opposed to Figure 4B, where the largest compound clusters make up the training set, leaving the singlet compounds and smallest clusters to make up the test set.

To optimize the hyperparameters for the realistic split models, we performed a 10‐fold cross‐validation. Linear sampling was used without shuffling the data, ordered by cluster number from largest to smallest. Therefore, with this method, each fold tested was the most dissimilar to the rest of the training set. The PCM models performed significantly worse on the realistic splits than on the randomly stratified splits (External test set *R* ^2^ Paired‐t(7) = 17.97; *p*‐value < 0.001—External test set RMSE Paired‐t(7) = 16.09; *p*‐value < 0.001) (Table 1). However, the metrics were still in the realms of powerful predictive ability, according to Grisoni et al. [27], having Q2 over 0.30. Overall, there were no significant differences between the algorithms used, namely RF and XGBoost.

Compared to the RDKit physicochemical descriptors, models trained with VSURF descriptors consistently performed worse on external sets (External test set *R* ^2^ Paired‐t(7) = 6.98; *p*‐value < 0.001) (Table 1). The difference in performance between the random and realistic split is noticeable when observing the physiochemical descriptors (RF: 0.77 vs. 0.53). More importantly, this drop in performance is also seen in the R2 values of the corresponding external test sets (RF: 0.78 vs. 0.51). When looking at the VSURF descriptors, the decrease in performance is only observed in the external test sets, not in cross‐validation (CV Q^2^ Paired‐t(7) = 0.03; *p*‐value = 0.98). This might be due to overfitting of the models trained with the VSURF descriptors and the better generalizability of the physiochemical descriptors.

Additionally, VSURF descriptors are expected to underperform classical 2D physiochemical descriptors since they are conformation‐dependent. In other words, while the dataset was energy minimized using the MMFF94x force field, it may not provide the bioactive conformation of the molecules in the dataset.

In recent years, PCM models have emerged in the literature as promising tools for identifying novel drug candidates for orphan targets [4], as is the case with GPCRs [28, 29]. Given the challenges associated with SLCs, as highlighted above, the potential of PCM in screening drug candidates for orphan SLC6 members becomes particularly intriguing. As part of our approach, we excluded one SLC6 member at a time during model training and focused on the remaining targets. This method was used to validate the model's ability to predict activities against the omitted target.

**FIGURE 5:**
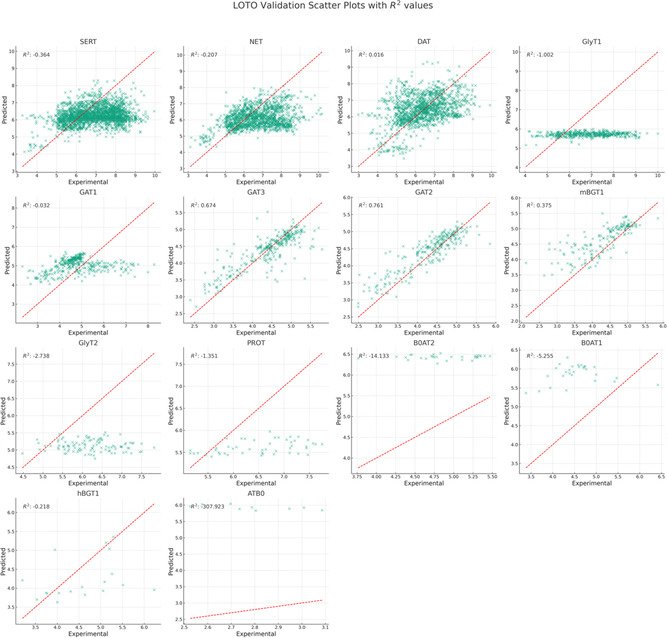
Leave One Target Out (LOTO) validation of the PCM models. Each scatter plot represents the external test set of the respective target using RF.

Interestingly, only activities tested on GAT2 and GAT3 were predicted with acceptable performance, namely, R2 of the test set is greater than 0.6, while LOTO validation on the rest of the SLC6 members was unsuccessful, as illustrated in Figure 5. GAT2 and GAT3 share a sequence identity above 93% in the binding pocket, which is all residues except for V122 in GAT2, corresponding to E140 in GAT3, as shown in Figure 1.

Moreover, GAT2 and GAT3 have 242 and 290 activity values in the dataset, respectively, 210 of which are identical molecules tested on both targets. This could explain why the model has performed so well on these two targets, as the chemical space of molecules and targets has been covered by the training set when testing both targets. Another challenge that hinders the success of LOTO validation in general is the high level of sparsity in the ligand x protein matrix (Figure S1). This translates to shifts in data distributions in the underlying data across different subfamilies, which may explain the forest averaging pattern in Figure 5. Therefore, in light of the current results, the PCM model is rendered incapable of providing reliable predictions of drug candidates for orphan SLC6 members in virtual screening scenarios.

By analyzing the built‐in measure of feature importance of the algorithms used to train the PCM models (Figure S2), it was apparent that one residue position ranked highest in all models across all splits, datasets, and descriptors, namely A173 in hSERT, corresponding to G149 and G153 in hNET and hDAT, respectively. While XGBoost has several feature importance metrics, we decided to focus on Gain and Total.

Gain as they relate to the features that most improved prediction accuracy. All indicators highlight the importance of the Z4 and Z2 descriptors of this position. While Z2 is associated with the volume and steric properties of amino acids, the interpretation of the Z4 descriptor is less interpretable [23]. Nevertheless, it corresponds to properties such as electronegativity and heat of formation [23].

This particular residue position in the MAT has been a topic of interest in various studies. Andersen et al. [30] introduced point mutations into the serotonin transporter to mimic the norepinephrine transporter and found that an A173G point mutation resulted in the largest change in the Ki of citalopram against its transporter hSERT, with a 14‐fold increase. Lynagh et al. [31] suggest that V148 and G149 in hNET corresponding to I172 and A173 in hSERT are the two amino acid differences in the substrate binding sites of NET and SERT that cause a low protein‐ligand complementarity. Goral et al. [32] proposed that residues V148 and G149 in hNET, corresponding to I172 and A173 in hSERT, are initial structural determinants of compound binding to hNET. In a further study by Tu et al. [33], it was reported that I172, A173, and L443 in hSERT and their corresponding residues in hNET and hDAT are the nonconserved “warm spot” binding site residues that play an important role in inhibitor selectivity in the MAT due to their different conformations.

Considering the GABA transporters subfamily, this position corresponds to a conserved asparagine residue that, to the best of our knowledge, does not seem to play a role in the binding of ligands to these transporters. However, this residue position could be important for the selectivity in the subfamily of MAT, considering the dataset imbalance. The dataset has a massive imbalance where more than 74% of the bioactivities belong to the MAT.

The second highest‐ranked residue position in the RF model was F341 in hSERT. While this residue is conserved across the MAT, it is a leucine residue in the GABA transporters, except for BGT1, where it is Q299. Recent studies have shown that Q299 is a key residue relevant to the activity and selectivity of BGT1 inhibitors [34].

To better understand how our models determine the importance of these descriptors, we visualized multiple trees from multiple models and observed one consistent pattern. The first decision made by a decision tree is always based on a protein descriptor. This could be explained by the fact that the different proteins in the training data have different distributions of molecular descriptors, and therefore, the data are split accordingly. An example decision tree is visualized in Figure S4.

## 4 Conclusions

In this study, we trained multiple PCM models using different data splits to investigate the potential for extrapolating chemical information through PCM. Our results show that train/test splits that only balance protein counts (target‐stratified split) lead to over‐optimistic model performance due to molecules experimentally tested on several targets, leading to identical (or highly similar) molecules being present in both the training and test sets. Given that PCM focuses on targets within the same family with high sequence identity and similar function, molecules tested on one target are often evaluated on others in the same subfamily. This results in many identical molecules being tested on multiple targets, leading to minimal chemical differences between the training and test sets if split randomly. Thus, a careful approach to data splitting is crucial to ensure the reliability and predictive accuracy of the model. Here, we implemented a realistic split that reduced the similarity between the training and test sets, which resulted in a more conservative model. We also explored the ability of PCM models to extrapolate chemical information to orphan targets using the LOTO split, with no success. This demonstrates a major limitation of PCM modeling in the extrapolation of information from data‐rich proteins to data‐scarce ones. Finally, our models further strengthen the experimentally derived evidence for the importance of residue position A173 in hSERT, corresponding to G149 and G153 in hNET and hDAT, respectively, to be relevant for subtype selectivity across the SLC6 family.

## Funding

This work was supported by the This study received funding from the RESOLUTE consortium. RESOLUTE has received funding from the Innovative Medicines Initiative 2 Joint Undertaking under grant agreement No 777372. This Joint Undertaking receives support from the European Union's Horizon 2020 research and innovation programme and EFPIA. This article reflects only the authors’ views and neither IMI nor the European Union and EFPIA are responsible for any use that may be made of the information contained therein (777372).

## Conflicts of Interest

The authors declare no conflicts of interest.

## Supporting information

Supplementary Material

## Data Availability

The data that support the findings of this study are available in ChEMBL at https://www.ebi.ac.uk/chembl/. These data were derived from the following resources available in the public domain: ChEMBL, https://www.ebi.ac.uk/chembl/.
