# Supplementary material for: To What Extent Can We Extrapolate Proteochemometric Models: A Case Study for the SLC6 Transporter Family
Source: Mol Inform. 2026 Jul 28;45(7):e70043. doi: 10.1002/minf.70043 (PMC13413613; doi:10.1002/minf.70043)
Supplement: Supplementary file 1 — Supplementary Material [file MINF-45-e70043-s001.pdf]

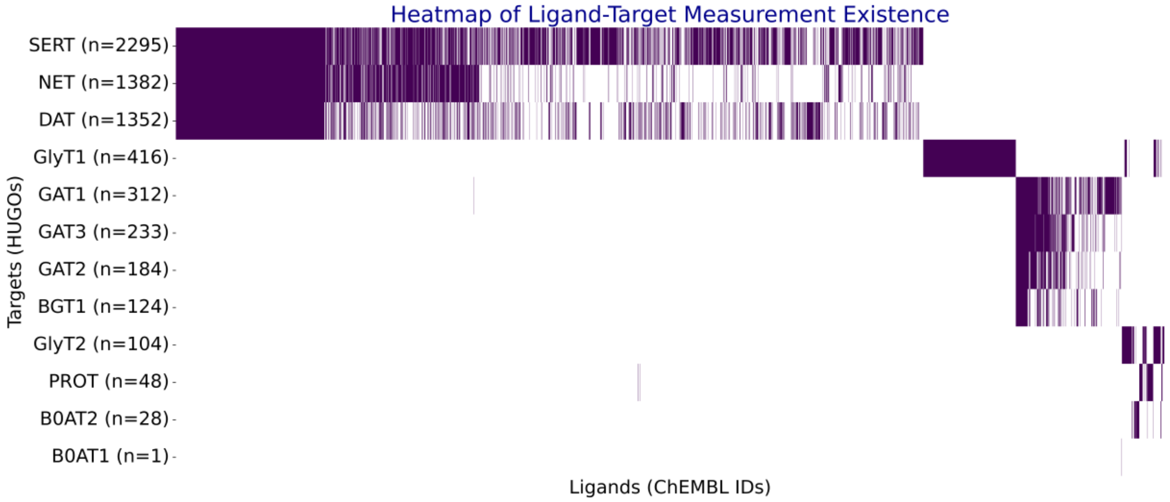

Figure S1. Visualization of ligand x target matrix of existing biological measurements. Notable overlap within subfamilies and non-existing overlap between subfamilies.

Table S1. Hyperparameter search grid for training the PCM models

Model	Hyperparameter Search Grid		
XGBoost		(Range)	(Step size)
	eta	0.01 – 0.41	0.05
	n_estimators	100 – 1000	100
	tree depth	5 – 30	5
RF	n_estimators	100, 200, 400, 800, 1500, 2000	
	max_depth	10, 30, 60, 80, None	
	max_features	“auto”, “sqrt”	
	min_samples_leaf	1, 2, 4	
	bootstrap	True, False	

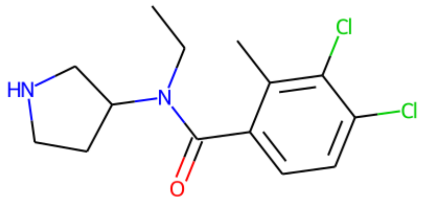

Figure S2. Example outlier (CHEMBL571536). NET (test set): measured pIC50 = 4.76, predicted = 7.62. Compound is present in training set against SERT: measured pIC50 = 8.0.

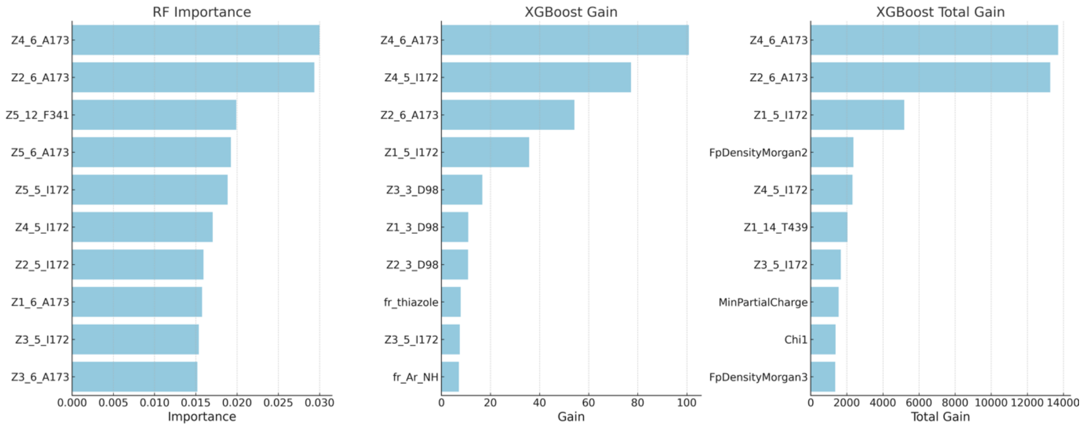

Figure S3. Feature importance analysis calculated by the built-in feature importance functions from RF and XGBoost.

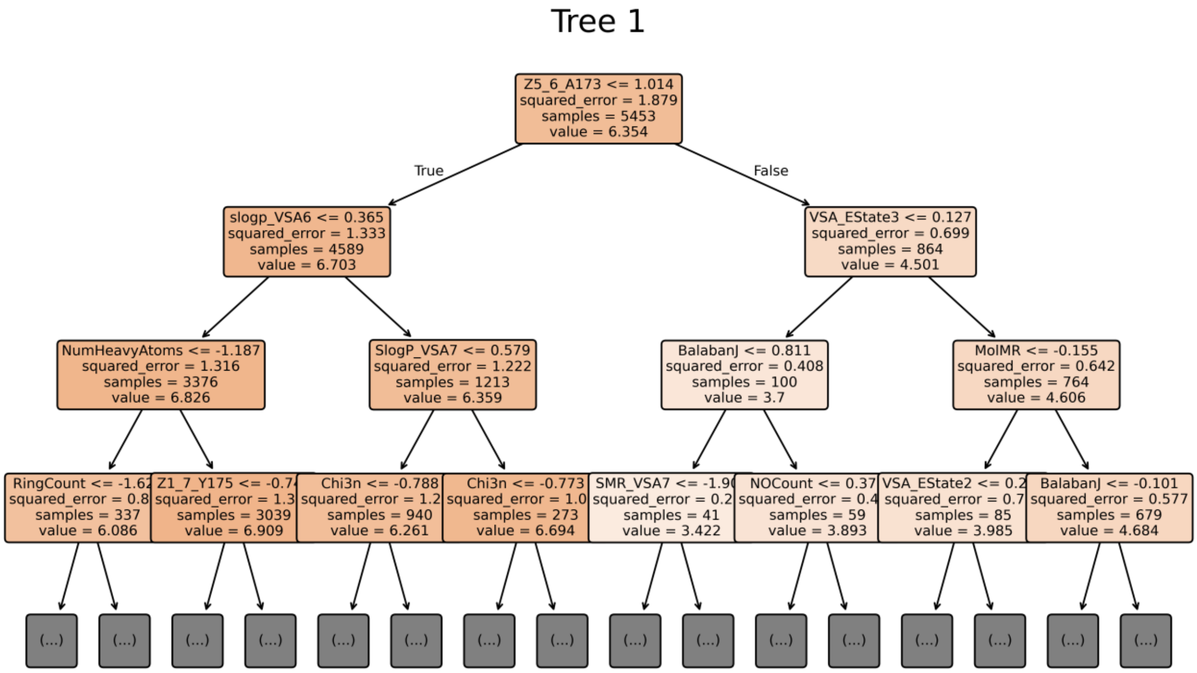

Figure S4. Example decision tree illustration. The first decision is based on protein descriptors, a consistent observation across all analyzed models and trees.
